# Time to acquire and lose carriership of ESBL/pAmpC producing *E. coli* in humans in the Netherlands

**DOI:** 10.1371/journal.pone.0193834

**Published:** 2018-03-21

**Authors:** Peter F. M. Teunis, Eric G. Evers, Paul D. Hengeveld, Cindy M. Dierikx, Cornelia C. C. H. Wielders, Engeline van Duijkeren

**Affiliations:** 1 Centre for Infectious Disease control, RIVM, Bilthoven, The Netherlands; 2 Hubert Department of Global Health, Rollins School of Public Health, Emory University, Atlanta GA, United States of America; University of Malaya Faculty of Medicine, MALAYSIA

## Abstract

A subset of the study population from a cross–sectional study of carriership of ESBL/pAmpC–producing *E. coli* (ESBL–E) in the general population was followed up by five successive samples over an approximate half year period, leading to six samples in 333 persons. Fecal samples were cultured and analyzed for the presence of *E. coli* types as characterized by MLST, and ESBL/pAmpC genes were analysed by PCR and sequencing. The study included 255 persons who had a negative first sample, to allow observations of acquiring carriership of ESBL–E. Any individual record thus consisted of a series of snapshots of episodes of presence and absence of ESBL–E carriage. A survival model was built to estimate times to acquire or lose carriership, allowing for any combination of ESBL/pAmpC gene and *E. coli* MLST type. In carriers, the mean time to lose carriership was 1.1 (95% range 0.8–1.6) years. The estimated mean time to acquire carriership was 3.0 (95% range 1.6–6.3) years. Analysis of these times by ESBL/pAmpC gene found substantial variation among resistance genes both in persistence of carriership and in rates of acquiring carriership: *bla*_CTX-M-1_, *bla*_CTX-M-14_, *bla*_CTX-M-15_, *bla*_CTX-M-27_ and *bla*_SHV-12_ were easily acquired, but *bla*_CTX-M-1_ and *bla*_SHV-12_ were also easily lost, while *bla*_CTX-M-15_, *bla*_CTX-M-27_ and *bla*_CMY-2_ were more likely to persist. When in addition bacterial host types were included, some combinations appeared more persistent than others (*bla*_CTX-M-1_ in ST10 and ST58; *bla*_CTX-M-14_, *bla*_CMY-2_, and *bla*_SHV-12_ in ST69), or were acquired with higher frequency (*bla*_CTX-M-14_ in ST38, ST69, and ST131; *bla*_CTX-M-15_ and *bla*_CTX-M-27_ in ST131; *bla*_SHV-12_ in ST69). The relatively short duration of carriership means that when an intervention drastically reduces the exposure of humans to ESBL-E, the prevalence will be halved in 0.66 years. The observed differences between carriage rates of ESBL/pAmpC genes and *E. coli* strains need further investigation.

## Introduction

Enterobacteriaceae with extended spectrum *β*–lactamase (ESBL) and/or plasmid–mediated AmpC (pAmpC) have been found not only in health–care settings, but also in the community at large. In a cross–sectional population study it was found that the prevalence of carriership of ESBL/pAmpC–producing Enterobacteriaceae in the community was 5.2% [1]. In a livestock–dense region a prevalence of 4.5% (ranging from 1.5 to more than 10%) was found [2], in accordance with other studies [3, 4].

These observed prevalences represent a balance between incident carriers, those acquiring ESBL/pAmpC–carrying enteric bacteria, and carriers who lose their resistant bacteria. Knowledge of the duration of carriership will be helpful to estimate the frequency of incident carriers needed to sustain the observed prevalences, which in turn is useful to understand the pressure exerted on the human population from different compartments of ESBL/pAmpC–producing Enterobacteriaceae: humans, livestock, food sources and the environment [2].

In order to study the duration of carriership, and the frequency with which carriership is acquired in the general population, subjects who had participated in the cross–sectional study [2] were invited to participate in a follow–up study, to collect longitudinal data on ESBL/pAmpC–producing *E. coli* and *K. pneumoniae*.

The present paper focusses on the development of a statistical method for analyzing the distributions of times spent in either of two states: presence or absence of carriership of ESBL/pAmpC–producing *E. coli* (ESBL–E).

Aside from the numbers of carriers in the population, it is important to identify heterogeneities. As various ESBL/pAmpC genes were detected in the cross–sectional population, it is possible to study whether these different genes have similar persistence in humans or not. Similarly, some resistance genes, or gene/bacterial host combinations may be more succesful than others in establishing carriership in humans.

## Materials and methods

### Data

**Fig 1:**
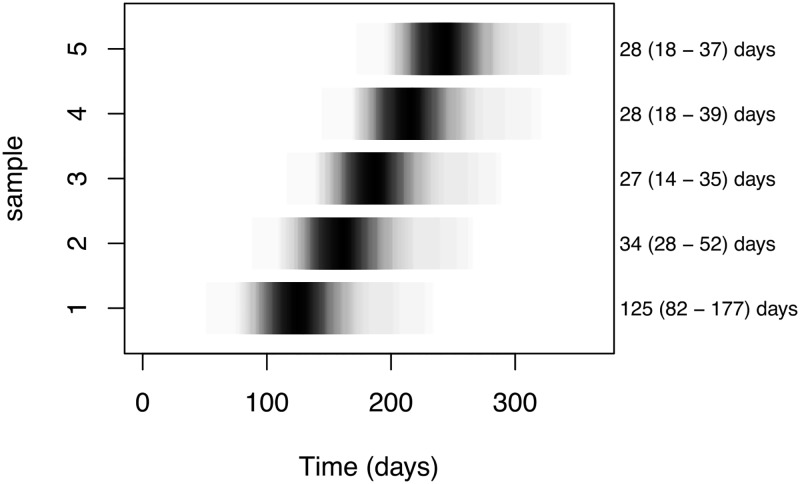
Sampling times. Distributions of sampling times, for each of the 5 samples taken after the first, cross–sectional sample (T = 0). The shading indicates quantiles of the distribution of sample times: darker means that the interval occurred more frequently. Note that the first interval exceeds 3 months duration, while the following intervals were close to the targeted duration of 1 month. Also, there was considerable variation in the lengths of sampling intervals (right hand numbers indicate median intervals and 95% range), resulting in overlapping intervals.

Data from a cross–sectional study of 2432 subjects randomly sampled from the adult population of a region with high density of animal farming [2] were used to select a cohort of 333 subjects. All 78 subjects who had a positive sample in the cross–sectional study were included, as well as an additional cohort of 255 subjects who had tested negative in the cross–sectional study. Each of the selected subjects was asked to submit five additional samples, targeted at (approximately) one month intervals, to have six successive samples over a half–year period (Fig 1).

Fecal samples were analyzed for the presence of ESBL–E, by selective enrichment followed by plating. Five colonies were selected for phenotypical detection of ESBL and/or pAmpC–producing *E. coli*, followed by identification of *bla*_CTX-M_ and/or *bla*_CMY_ and *bla*_DHA_ *β*–lactamase genes and MLST typing of bacteria strains. Details of laboratory methods can be found in [2].

S1 Table lists the genes that were identified, S2 Table lists all *E. coli* strains identified by MLST typing. Note that any samples that were analyzed for the presence of ESBL–E, may contain multiple resistance genes in any number of bacterial strains.

Table 1 shows a sample fragment of the data. For any subject, the goal was six successive samples, but some were missing, resulting in a smaller number of samples. In case resistant bacteria were found, up to five different colonies were excised and analyzed to identify ESBL/pAmpC genes and MLST types of their bacterial hosts. All persons with a sample at time point 0 and at least one additional sample were included.

**Table 1:** Data fragment.

subj.	time	time	time	time	time	time
ID	0	1	2	3	4	5
1	410/CTX-M-8	-	-	-	-	-
2			NA			69/CTX-M-1
				218/CTX-M-1		
					399/CTX-M-1	
	1250/CTX-M-1					
		1277/CTX-M-1		1277/CTX-M-1		1277/CTX-M-1
3	-	-	-	-	-	-
4	131/CTX-M-15	131/CTX-M-15	131/CTX-M-15	131/CTX-M-15	131/CTX-M-15	131/CTX-M-15
5	-	131/CTX-M-15	131/CTX-M-15	131/CTX-M-15	131/CTX-M-15	
	-					2451/CTX-M-15
6	-	NA	-	NA	NA	NA

Fragment of the data showing subject ID and status of up to 5 MLST type/resistance gene combinations in cultured samples at times 0, 1, …, 5. Each type detected once in a person is shown on a separate line. Samples may be missing (NA), or negative (-) indicating that no ESBL/pAmpC genes were found.

### Methods

As described above, longitudinal data collected in this study consisted of six successive samples, at intervals of about a month, except for the first interval that was longer (Fig 1). For each subject there is a sequence of carrier status information: date of sampling, zero or more ESBL/pAmpC genes, and zero or more MLST types of bacterial hosts. In the present analysis only *E. coli* strains were used.

Carrier status thus is known from observations at discrete times, and any changes in carrier status, occurring between samples, cannot be directly observed. Methods for dealing with longitudinal observations of subjects where their states are observed at discrete times were developed for estimating transition times in AIDS [5–7]. Recently, similar methods have been applied to carriership of resistant microbes [8].

#### Waiting times between states

**Fig 2:**
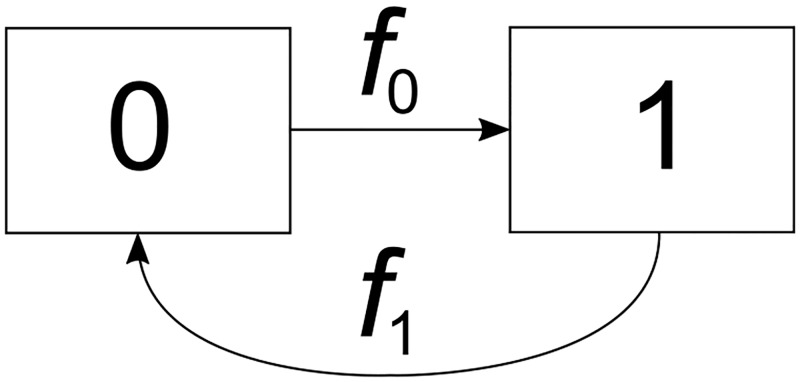
Carrier states. State diagram of the two possible states and transitions among these states. The waiting time *τ* in state 0 has probability density *f*_0_(*τ*) and in state 1 the waiting time distribution is *f*_1_(*τ*).

Using the framework proposed by [5], an individual can be in two states: negative (0) and positive (1), Fig 2. The waiting time *τ* in state 0 has probability density *f*_0_(*τ*), the waiting time in state 1 has density *f*_1_(*τ*).

**Fig 3:**
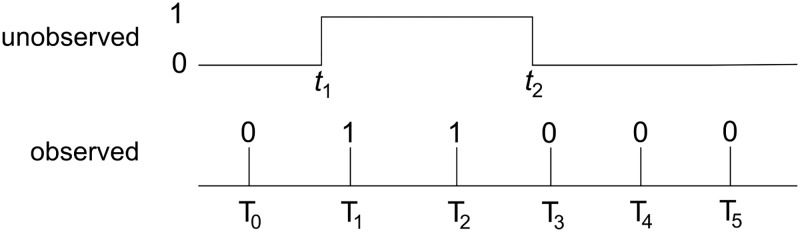
Time course. A hypothetical time course where a host becomes positive at (unobserved) time *t*_1_, after having become negative at some time (*t*_0_) before the first observation, and returns to negative status at time *t*_2_. Observations occur at times *T*_0_, *T*_1_, *T*_2_, *T*_3_, *T*_4_, and *T*_5_, with outcomes 0, 1, 1, 0, 0, and 0 respectively.

The first observation is made at time *T*_0_ and the last observation is made at time *T*_5_. Suppose the time course of the state of this subject is as shown in Fig 3: the sample at *T*_0_ is negative, the samples at *T*_1_ and *T*_2_ are positive, and the last three samples (*T*_3_, *T*_4_, *T*_5_) are all negative. We assume that a state change 0 → 1 occurred between *T*_0_ and *T*_1_ at some unobserved time *t*_1_. Another state change 1 → 0 occurred between *T*_2_ and *T*_3_, at time *t*_2_, also unobserved.

During the interval *T*_0_ → *t*_1_ the subject is in state 0 but may have entered this state anytime before *T*_0_, thus the first episode is censored: *τ* ∈ (*t*_1_ − *T*_0_, ∞). During the second episode *t*_1_ → *t*_2_ the subject is positive (state 1), exactly between *t*_1_ and *t*_2_ (uncensored). During the third interval *t*_2_ → *T*_5_ the subject is again in state 0 but may remain there after *T*_5_, this interval is again censored: *τ* ∈ (*T*_5_ − *t*_2_, ∞). The transition times *t*_1_ and *t*_2_ have not been observed but it is known that *t*_1_ ∈ (*T*_0_, *T*_1_] and *t*_2_ ∈ (*T*_2_, *T*_3_].

The likelihood function for the observed sequence in Fig 3 becomes

$$\begin{array}{c} \ell \left(t_{1},t_{2}\right)=\int_{\tau =t_{1}-T_{0}}^{\infty }f_{0}\left(\tau \right)\text{d}\tau \times f_{1}\left(t_{2}-t_{1}\right)\times \int_{\tau =T_{5}-t_{2}}^{\infty }f_{0}\left(\tau \right)\text{d}\tau \end{array}$$ (1)

Or, in terms of the cumulative distribution functions of the waiting times

$$\begin{array}{c} \ell \left(t_{1},t_{2}\right)=(1-F_{0}\left(t_{1}-T_{0}\right))\times f_{1}\left(t_{2}-t_{1}\right)\times (1-F_{0}\left(T_{5}-t_{2}\right)) \end{array}$$ (2)

The transition times *t*_1_ and *t*_2_ are unobserved, but since *T*_0_ < *t*_1_ < *T*_1_ and *T*_2_ < *t*_2_ < *T*_3_, the marginal likelihood can be calculated

$$\begin{array}{c} L=\int_{t_{1}=T_{0}}^{T_{1}}\int_{t_{2}=T_{2}}^{T_{3}}\ell \left(t_{1},t_{2}\right)\text{d}t_{1}\text{d}t_{2} \end{array}$$ (3)

Note that this means that the data are doubly censored: state changes can only be observed at the start or end of an interval, and any events leading to the initial state or events ending the last observed state remain unobserved.

Using the above framework, the likelihood function for an arbitrary sequence of longitudinal samples may be worked out, by

1. accounting for left censoring: the contribution of the inital state of the subject to the likelihood function
2. detecting transitions (0 → 1 and 1 → 0)
3. collecting likelihood contributions for each transition
4. accounting for right censoring: the contribution of the final state of the subject to the likelihood function

#### Parametric model for waiting time distributions

A convenient parametric distribution often used for modelling failure rates is the Weibull distribution [9]. We assume that the waiting time distributions, both for acquiring carriership and for loosing carriership, are Weibull distributions with density

$$\begin{array}{c} f(\tau |r,\lambda )=\left(\frac{r}{\lambda }\right){\left(\frac{\tau }{\lambda }\right)}^{r-1}{\text{e}}^{-{\left(\tau /\lambda \right)}^{r}} \end{array}$$ (4)

and cumulative distribution function

$$\begin{array}{c} F\left(\tau |r,\lambda \right)=\int_{u=0}^{\tau }f\left(u|r,\lambda \right)\text{d}u=1-{\text{e}}^{-{\left(\tau /\lambda \right)}^{r}} \end{array}$$ (5)

where λ is a scale parameter for the waiting time, and the shape parameter *r* indicates how the failure rate or hazard changes over time (*r* < 1 decrease; *r* > 1 increase; when *r* = 1 the hazard does not change over time). Compared to a simpler model with constant hazards, leading to exponentially distributed waiting times, the shape parameter *r* may be adjusted to values greater than 1, to prevent implausibly short waiting times. As observations are typically 1 month apart, rapid changes in carriership status could not be observed. Using a suitably crafted prior forcing *r* > 1 prevents occurrence of multiple changes of carriership between observations.

Using Eq (2), the likelihood function for the transition times *t*_1_ and *t*_2_ in the example in Fig 3 then becomes

$$\begin{array}{c} l\left(t_{1},t_{2}\right)={\text{e}}^{-{\left(\frac{t_{1}-T_{0}}{\lambda_{0}}\right)}^{r_{0}}}\left(\frac{r_{1}}{\lambda_{1}}\right){\left(\frac{t_{2}-t_{1}}{\lambda_{1}}\right)}^{r_{1}-1}{\text{e}}^{-{\left(\frac{t_{2}-t_{1}}{\lambda_{1}}\right)}^{r_{1}}}\times {\text{e}}^{-{\left(\frac{T_{5}-t_{2}}{\lambda_{0}}\right)}^{r_{0}}} \end{array}$$ (6)

where λ_0_ and *r*_0_ describe the waiting time distribution for acquiring carriership, and λ_1_ and *r*_1_ the waiting time distribution for losing carriership.

#### Numerical evaluation

Instead of evaluating the integration in Eq (3) the likelihood can be calculated in a MCMC sampler, accounting for the censored observations of the transition times. In the above example these are the times *t*_1_ and *t*_2_.

Samples may be missing, resulting in fewer than six samples, and correspondingly longer intervals, unless the last sample is missing. The first sample is not missing, obviously.

In order to limit the number of free parameters, the Weibull shape parameters *r*_0_ and *r*_1_ were assumed fixed among ESBL/pAmpC genes and/or *E. coli* strains. The difference between strata (ESBL/pAmpC genes, *E. coli* strains or combinations) were assumed scale differences, per scale parameter λ_0_ or λ_1_.

The two parameters *r* and λ define the distributions of the times to acquire or lose carriership. Given estimates for (*r*, λ), the mean time to change of carriership status (acquire or lose carriership) can be calculated:

$$\begin{array}{c} E\left(\tau \right)=\lambda \Gamma \left(1+\frac{1}{r}\right) \end{array}$$

the median time is:

$$\begin{array}{c} Q_{0.5}\left(\tau \right)=\lambda {\left(\text{log}\;2\right)}^{\frac{1}{r}} \end{array}$$

Other statistics may be calculated also (like variance, or 95% predictive interval).

The model was implemented in JAGS v4.2.0 [10], called from R v3.3.2 [11] using the rjags package v4-6 [12]. Source code is provided in the appendix.

#### Analysis by resistance gene and/or bacterial host strain

Positive observations may include a variety of resistance genes, possibly different in any of the (6) samples of an individual during the longitudinal study. Suppose a longitudinal set of samples for a subject results in 0 1 1 0 1 0 (0 for absence, 1 for carriership of resistance). The positive samples may contain any resistance genes from a set of detected genes, say (*a*, *b*, *c*, *d*). Including these resistance genes the above set of samples may contain

$$\begin{array}{c} 0(\begin{array}{c} a\\ b \end{array})c\;0(\begin{array}{c} a\\ b\\ c \end{array})0 \end{array}$$

for instance: the first sample is negative; in the second sample both *a* and *b* are found, in the third sample only *c* is found, the fourth sample is negative, the fifth contained *a*, *b* and *c*, and the last sample was again negative. This may be read, as the following set of positive/negative sequences

$$\begin{array}{c} \begin{array}{c} a\;0\;1\;0\;0\;1\;0\\ b\;0\;1\;0\;0\;1\;0\\ c\;0\;0\;1\;0\;1\;0\\ d\;0\;0\;0\;0\;0\;0 \end{array} \end{array}$$

for each of the genes in the set (*a*, *b*, *c*, *d*). These sequences provide information about the occurrence of carriership of each distinct gene separately.

If carriage of one gene *a* is independent of carriage of a different gene *b*, then the likelihood for the joint pattern for all observed genes becomes the product of the likelihood of all separate sequences. In case these are *K* genes, numbered 0, 1, 2, …, *K* each with a contribution ${ℒ}_{k}$ the joint likelihood is

$$\begin{array}{c} L=\prod_{k=1}^{K}L_{k} \end{array}$$ (7)

The same reasoning applies when analyzing carriage times by MLST type of the bacterial host cells, or combinations of host type and ESBL/pAmpC gene.

In case carriage of different genes/host strains can not be considered independent, these likelihood contributions must be conditioned on the combinations of concurrently carried genes. Such dependence is not considered in this paper, as it complicates analysis, but more importantly because carriage is relatively rare, so that concurrent carriage is not observed abundently.

## Results

When resistance gene or *E. coli* strain is ignored, and only presence or absence of any *E. coli* strain carrying a ESBL/pAmpC gene is considered, the time to acquire carriership and the time to lose carriership can be estimated.

**Fig 4:**
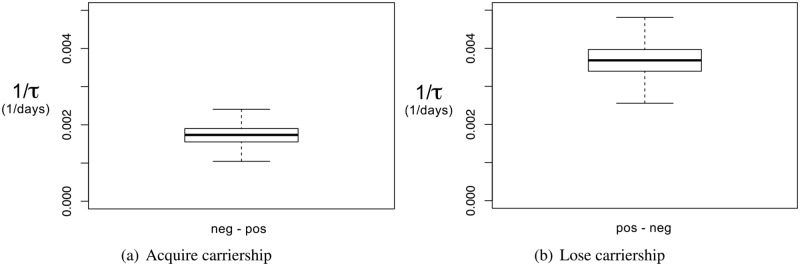
Rates of state change. Estimates of the rates for state change 0 → 1 (acquire carriership) and 1 → 0 (lose carriership). Carriers shed any ESBL/pAmpC gene in any *E. coli* host.

Estimates of the Weibull parameters are given in Table 2. From these parameter estimates, statistics of the time to carriership change can be calculated. The median time to acquire carriership is approximately 3.0 years, with a range from 1.6 to 6.3 years. The median time to lose carriership is 1.1 years, ranging from 0.8 to 1.6 years (see top two rows in Table 2). In order to compare estimates it is convenient to calculate frequencies of acquiring or losing carriership, so that long times can be shown as near–zero rates. Fig 4 shows the frequencies of acquiring and losing carriership, of any ESBL/pAmpC gene: at these low rates this is approximately the daily probability of acquiring or losing carriership.

**Table 2:** Parameter estimates.

	*r*			λ (days)			*E*(*τ*) (days)		
	*P*_50_	*P*_2.5_	*P*_97.5_	*P*_50_	*P*_2.5_	*P*_97.5_	*P*_50_	*P*_2.5_	*P*_97.5_
Carriage of any ESBL/pAmpC gene/bacterial host strain									
acquire	2.0	1.4	2.7	1.2×10^3^	0.7×10^3^	2.5×10^3^	1.1×10^3^	0.6×10^3^	2.3×10^3^
lose	1.2	0.9	1.6	0.4×10^3^	0.3×10^3^	0.6×10^3^	0.4×10^3^	0.3×10^3^	0.6×10^3^
By ESBL/pAmpC gene in any bacterial host strain									
acquire									
*bla*_CTX-M-1_	1.5	1.1	2.1	0.8×10^4^	0.2×10^4^	6.1×10^4^	0.7×10^4^	0.2×10^4^	5.7×10^4^
*bla*_CTX-M-15_	1.5	1.1	2.1	0.5×10^4^	0.2×10^4^	2.2×10^4^	0.4×10^4^	0.2×10^4^	2.1×10^4^
*bla*_CTX-M-14_	1.5	1.1	2.1	0.5×10^4^	0.2×10^4^	2.3×10^4^	0.4×10^4^	0.2×10^4^	2.2×10^4^
*bla*_CTX-M-27_	1.5	1.1	2.1	0.5×10^4^	0.2×10^4^	2.2×10^4^	0.4×10^4^	0.2×10^4^	2.1×10^4^
*bla*_SHV-12_	1.5	1.1	2.1	1.5×10^4^	0.3×10^4^	32.0×10^4^	1.4×10^4^	0.3×10^4^	30.4×10^4^
*bla*_CMY-2_	1.5	1.1	2.1	1.7×10^7^	1.3×10^4^	5.9×10^14^	1.5×10^7^	1.2×10^4^	5.4×10^14^
lose									
*bla*_CTX-M-1_	1.2	0.9	1.4	1.8×10^2^	0.9×10^2^	4.7×10^2^	1.7×10^2^	0.8×10^2^	4.6×10^2^
*bla*_CTX-M-15_	1.2	0.9	1.4	4.1×10^2^	2.6×10^2^	7.8×10^2^	3.9×10^2^	2.4×10^2^	7.6×10^2^
*bla*_CTX-M-14_	1.2	0.9	1.4	5.9×10^2^	2.8×10^2^	19.5×10^2^	5.6×10^2^	2.6×10^2^	19.0×10^2^
*bla*_CTX-M-27_	1.2	0.9	1.4	4.0×10^2^	1.7×10^2^	14.8×10^2^	3.9×10^2^	1.6×10^2^	14.6×10^2^
*bla*_SHV-12_	1.2	0.9	1.4	1.2×10^2^	0.6×10^2^	3.2×10^2^	1.2×10^2^	0.6×10^2^	3.0×10^2^
*bla*_CMY-2_	1.2	0.9	1.4	3.1×10^2^	1.3×10^2^	10.9×10^2^	2.9×10^2^	1.2×10^2^	10.6×10^2^

Parameter estimates for the Weibull distributions of times to acquire and lose carriership. The shape parameter *r* is assumed the same for all strata (ESBL/pAmpC genes); estimates of the scale parameter λ may be stratified by ESBL/pAmpC gene. Also shown is the mean time to acquire or lose carriership (*E*(*τ*)). For all estimates, median values (*P*_50_) and 95% predictive ranges (*P*_2.5_–*P*_97.5_) are given, to illustrate uncertainty.

S1 Fig shows the shape of the Weibull distributions of the times to acquire and lose carriership. S7 Fig shows 95% contours for the posterior distribution of the parameters (*r*_0_, λ_0_) and (*r*_1_, λ_1_).

Although 17 different ESBL/pAmpC genes were found in the longitudinal study (see S1 Table), only the 6 most frequently found genes were considered here: *bla*_CTX-M-1_, *bla*_CTX-M-14_, *bla*_CTX-M-15_, *bla*_CTX-M-27_, *bla*_SHV-12_, and *bla*_CMY-2_. Any other resistance gene has been designated “other”.

**Fig 5:**
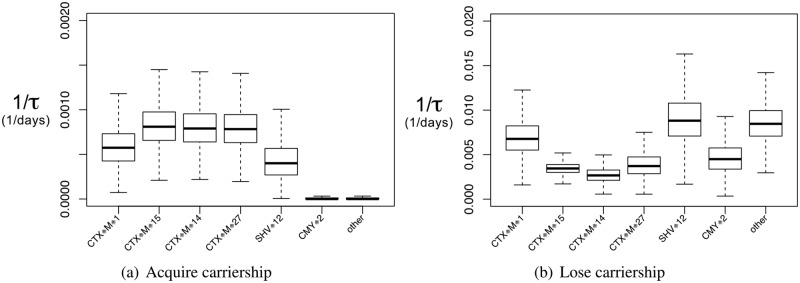
Rates of state change by gene. Estimates of the rates for state change 0 → 1 (acquire carriership) and 1 → 0 (lose carriership), by ESBL/pAmpC gene in any *E. coli* host, among the longitudinal study participants. Only the most frequent ESBL/pAmpC genes are shown. Note the different scales for acquirement and loss of carriership.

Parameter estimates and average times (to acquire and lose carriership) are listed in Table 2. The estimated rates for acquiring carriership are different among the 7 ESBL/pAmpC categories. For *bla*_CTX-M-1_, *bla*_CTX-M-14_, *bla*_CTX-M-15_, *bla*_CTX-M-27_, and *bla*_SHV-12_ the rates are 0.0003 days^−1^ or higher (waiting time 9 years or shorter), while the rates for all other gene types are much smaller (Fig 5).

Among the estimated rates for losing carriership, the pattern is different. Here, *bla*_CTX-M-1_ and *bla*_SHV-12_ have high rates above 0.005 days^−1^ (waiting time shorter than 0.5 years), while the rates of losing carriership of *bla*_CTX-M-14_, *bla*_CTX-M-15_, *bla*_CTX-M-27_, and *bla*_CMY-2_ are approximately half as high.

Overall, rates for losing carriership are higher than those for acquiring carriership, as in the above analysis that ignored resistance gene type. Note the vertical axes of the two graphs. The estimated rate of losing carriership in the category “other” also was high, indicating that there too, genes are lost more easily than they are acquired.

Corresponding distributions for times to acquire or lose carriership can be found in S2 and S3 Figs.

Combining resistance genes and genotype of the detected resistant *E. coli*, estimates could be found for 20 combinations of ESBL gene and MLST type (S3 Table).

**Fig 6:**
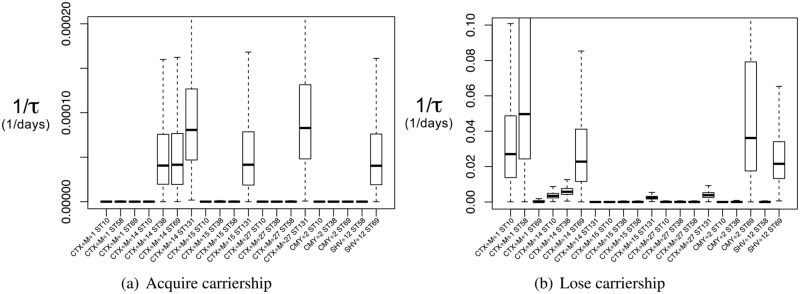
Rates of state change by gene and strain. Estimates of the rates for state change 0 → 1 (acquire carriership) and 1 → 0 (lose carriership) by ESBL/pAmpC gene–*E. coli* MLST type combination, among the longitudinal study participants. Note the different scales for acquirement and loss of carriership.

As can be seen in Fig 6, some combinations have much higher estimated rates than others. Acquiring *bla*_CTX-M-14_ occurs frequently in ST38, ST69, and ST131 (but not in ST10); *bla*_CTX-M-15_ in ST131 (but not in ST10, ST38, and ST58); *bla*_CTX-M-27_ in ST131 (but not in ST10, ST38, ST58); and *bla*_SHV-12_ in ST69 (but not in ST58). Parameter estimates and average times to acquire carriership can be found in S4 Table.

Losing carriership occurs faster for *bla*_CTX-M-1_ in ST10 and ST58 (but not in ST69); *bla*_CTX-M-14_ in ST69 (and less in ST10, ST38, and not in ST131); *bla*_CMY-2_ in ST69 (and not in ST10 and ST38); and *bla*_SHV-12_ in ST69 (and not in ST58). Parameter estimates and average times can be found in S5 Table.

## Discussion

This longitudinal study aimed to investigate duration of carriage of extended–spectrum *β*–lactamase and pAmpC *β*–lactamase–producing *Escherichia coli* in the community. The present study is unique in that ESBL-E negative subjects were included, allowing observations of acquiring carriership: transitions from a negative to a positive state. Generally, longitudinal studies are scarce and most studies focus on hospital patients or travellers, while our study investigated individuals in the general population in a livestock–dense area. The follow–up data collected in the longitudinal study provide rich information on sequences of carriership in individual subjects. With six successive time points there is ample information on the time course of ESBL–E carriership, and by analyzing five different colonies from each cultured sample, concurrent carriage of multiple genes and MLST types is likely to be detected (compared to when a single colony is used for sequencing).

Overall, ignoring gene types and bacterial host strains, the rates of losing carriership are higher than the rates of acquiring carriership. The estimated overall mean time to lose carriership (1.1 yr, 95% range 0.8–1.6 yr) is in agreement with reported decrease in fecal carriage after urinary tract infection [13]. Similar rates were estimated in a household transmission study [14], for index patients. It may be noted that, in case an intervention would be successful enough to completely prevent new carriers from occurring, the prevalence of carriership would decrease (approximately) exponentially with a halftime of 0.66 years (using simulated waiting times from the fitted Weibull distribution for the unstratified model).

The estimated mean time to acquire carriership is longer (3.0 yr, 95% range 1.6–6.3 yr). Carriership is more rapidly lost than it is acquired, which is to be expected given the observed prevalence of 5%.

Overall only 15% of all samples collected at any time during the longitudinal study tested positive for any ESBL/pAmpC type. Even in this population selected for a high proportion of carriers, 66% of individual records contained only negative samples. As discussed above (Methods section), separation by gene and/or strain dilutes the data, leaving multiple sequences with sparse information on the time course of carriership of specific genes/strains. The method proposed in this study allows inference for such sparse data, using information of on→off (79 observed) and off→on (45 observed) sequences. Only when available records do not include sufficient numbers of on→off or off→on sequences, uncertainty becomes dominant. This may be appreciated by comparing estimated distributions for *bla*_CMY-2_ and *bla*_SHV-12_ in S2 Fig: the shading indicates that *bla*_CMY-2_ estimates are highly uncertain.

Compared to the general population, losing carriership in the study population may be unbiased (or not strongly biased). Persistence of ESBL–E with the same strain/gene (given that a person is a carrier) may not depend strongly on host behaviour. It is however easy to see how rates of acquiring carriership may depend more strongly on (behaviour of) the sampled population. Assuming steady state, the rate estimates for acquiring and losing carriership would lead to high prevalence (around 35%), compared to observed numbers in the cross–sectional population [2]. For the longitudinal study, subjects who were positive in the initial sample (i.e. the 4.5% positives in the cross–sectional sample) were overrepresented in the longitudinal sample (23% with initial sample positive). This is by design, in order to avoid having very few longitudinal samples positive. The longitudinal cohort may therefore have included a greater proportion of subjects with increased risk of ESBL-E carriership, resulting in underestimation of the time to acquire carriership. Increased risk of ESBL–E carriage may have been associated with microbial factors, like high infectivity of the *E. coli* strains and/or ESBL/pAmpC genes found in the longitudinal cohort. Host factors like exposure behaviour, susceptibility to infection, and specific factors like use of antimicrobials may also have been different from the general population. As shown recently, *E. coli* phylogenetic group B2 and D and travel to ESBL–high–prevalence countries are associated with prolonged carriage in this same cohort [15].

This analysis assumes all observed sequences are without measurement error. There may have been false negatives. Even though selective enrichment and culturing were used, low numbers of bacteria carrying resistance genes may have been missed. Thus, when a sequence includes a positive sample followed by a negative sample which is then followed again by a positive sample, for the same gene, the intermediate negative may have been a false negative. Assayed samples may also miss information on variation in ESBL genes/E. coli strains, leading to underestimation of diversity in the sample.

Interestingly, analysis by gene and by gene/bacterial host combination reveals marked differences in rates of acquiring and losing carriership. Some of these estimates are based on small numbers of observations, as is clear from inspecting the uncertainty, e.g. in S2 Fig Most of these differences, however, are based on sufficient observations and may be assumed to reflect “true” variation in the frequency of acquiring carriership and its persistence. Generally, isolates carrying *bla*_CTX-M-1_, *bla*_CTX-M-14_, *bla*_CTX-M-15_, *bla*_CTX-M-27_ and *bla*_SHV-12_ were easily acquired compared to *bla*_CMY-2_, while isolates carrying *bla*_CTX-M-14_, *bla*_CTX-M-15_ and *bla*_CTX-M-27_ persisted longer than isolates carrying *bla*_CTX-M-1_ or other genes. As mentioned earlier, the rate of acquiring carriership may reflect properties of the gene–plasmid combination or its bacterial host, but also exposure behaviour of the human carrier, like travelling, or antimicrobial usage. The rate of losing carriership may also reflect gene, plasmid, or bacterial host properties that determine persistence, but also the competence of the human carrier to clear the carriage of the resistant *E. coli* strain or the resistance gene.

In view of this difference in causation, it is interesting that a preliminary check indicates that resistance gene type and *E. coli* strain seem to act independently in acquiring carriership, but that several combinations of ESBL/pAmpC gene and *E. coli* MLST type appear to be more persistent than would be predicted by their separate rates (of losing carriership). See S1 Appendix. To shed more light on the problem of whether transfer of resistance genes between bacterial hosts is horizontal or clonal, it would therefore be interesting to study correlation between acquiring and/or losing different genes and/or bacterial strains carrying these genes.

For better understanding of the transmission of ESBL/pAmpc resistance, the model may be adapted to study how the rate estimates may depend on characteristics of the human carrier: age, gender, and specific risk factors like antibiotic usage. The present paper aims at presenting the analysis method and establishing that it can be used to estimate rates of acquiring and losing carriership. Study of covariables of the carrier population is subject of a separate paper following up on the present study.

## Conclusion

In conclusion, the estimated time to lose carriership (mean time approximately 400 days) indicates that any intervention that decreases exposure is expected to take about a year to result in decreased prevalence (halftime 0.66 years). The differences between the time to acquire and lose certain ESBL/pAmpC genes and *E. coli* strains needs further investigation.

## Supporting information

S1 Fig (PDF)

S2 Fig (PDF)

S3 Fig (PDF)

S4 Fig (PDF)

S5 Fig (PDF)

S6 Fig (PDF)

S7 Fig (PDF)

S1 Table (PDF)

S2 Table (PDF)

S3 Table (PDF)

S4 Table (PDF)

S5 Table (PDF)

S1 Appendix (PDF)

S2 Appendix (PDF)

S3 Appendix (PDF)
