# Correction: Time to acquire and lose carriership of ESBL/pAmpC producing *E*. *coli* in humans in the Netherlands

**DOI:** 10.1371/journal.pone.0196492

**Published:** 2018-04-23

**Authors:** Peter F. M. Teunis, Eric G. Evers, Paul D. Hengeveld, Cindy M. Dierikx, Cornelia C. H. Wielders, Engeline van Duijkeren

The fifth author’s name is incorrect. The correct name is: Cornelia C. H. Wielders. The correct citation is: Teunis PFM, Evers EG, Hengeveld PD, Dierikx CM, Wielders CCH, van Duijkeren E (2018) Time to acquire and lose carriership of ESBL/pAmpC producing *E*. *coli* in humans in the Netherlands. PLoS ONE 13(3): e0193834. https://doi.org/10.1371/journal.pone.0193834
