# Supplementary material for: Time to acquire and lose carriership of ESBL/pAmpC producing E. coli in humans in the Netherlands
Source: PLoS One. 2018 Mar 21;13(3):e0193834. doi: 10.1371/journal.pone.0193834 (PMC5862452; doi:10.1371/journal.pone.0193834)
Supplement: S1 Fig — (PDF) [file pone.0193834.s001.pdf]

S1 Fig. Waiting time distributions: unstratified

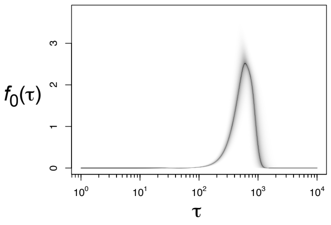

(a) Acquire carriership

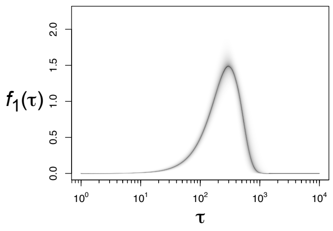

(b) Lose carriership

Distribution of the waiting time (days) for state change $0 \rightarrow 1$ (acquire carriership) and $1 \rightarrow 0$ (lose carriership). Carriers shed any ESBL/pAmpC gene in any *E. coli* host. Note that these are density graphs showing the shape of the fitted Weibull distributions; the shading illustrates uncertainty (where the median is dark, and decreasing shading intensity at lower and higher quantiles). Also note that the time scale is logarithmic.
