# Supplementary material for: Time to acquire and lose carriership of ESBL/pAmpC producing E. coli in humans in the Netherlands
Source: PLoS One. 2018 Mar 21;13(3):e0193834. doi: 10.1371/journal.pone.0193834 (PMC5862452; doi:10.1371/journal.pone.0193834)
Supplement: S2 Fig — (PDF) [file pone.0193834.s002.pdf]

S2 Fig. Waiting time distributions: acquiring carriership by ESBL/pAmpC gene

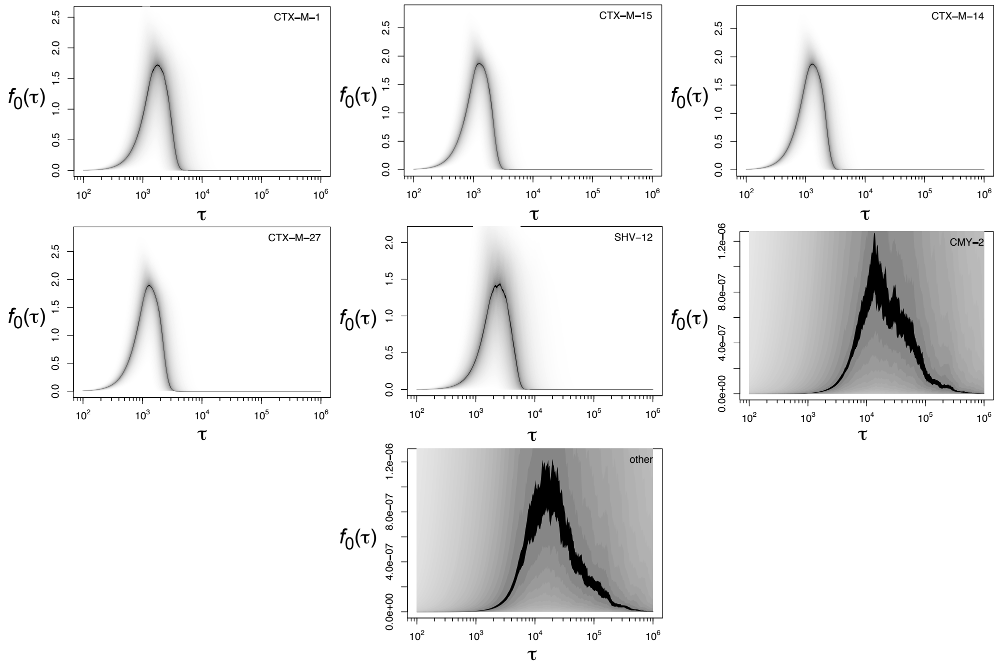

Distribution of the waiting time for state change $0 \to 1$ (acquire carriership) by ESBL/pAmpC gene, in any *E. coli* host.
