# Supplementary material for: Time to acquire and lose carriership of ESBL/pAmpC producing E. coli in humans in the Netherlands
Source: PLoS One. 2018 Mar 21;13(3):e0193834. doi: 10.1371/journal.pone.0193834 (PMC5862452; doi:10.1371/journal.pone.0193834)
Supplement: S3 Fig — (PDF) [file pone.0193834.s003.pdf]

S3 Fig. Waiting time distributions: losing carriership by ESBL/pAmpC gene

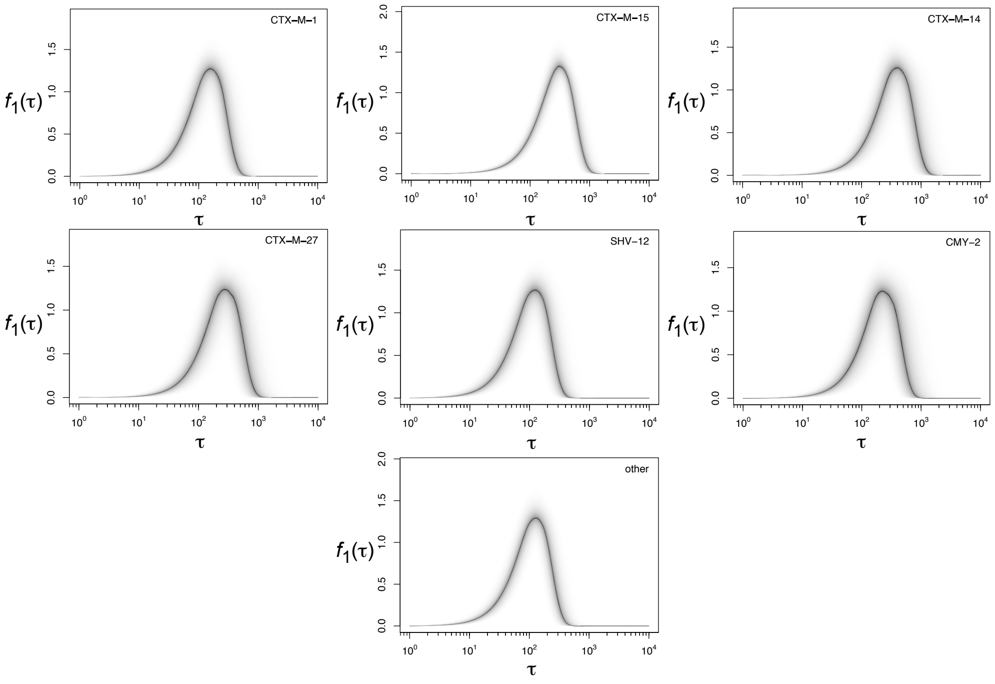

Distribution of the waiting time for state change $1 \rightarrow 0$ (lose carriership) by ESBL/pAmpC gene, in any *E. coli* host.
