# Supplementary material for: Time to acquire and lose carriership of ESBL/pAmpC producing E. coli in humans in the Netherlands
Source: PLoS One. 2018 Mar 21;13(3):e0193834. doi: 10.1371/journal.pone.0193834 (PMC5862452; doi:10.1371/journal.pone.0193834)
Supplement: S4 Fig — (PDF) [file pone.0193834.s004.pdf]

S4 Fig. Rates of state change by ESBL/pAmpC gene

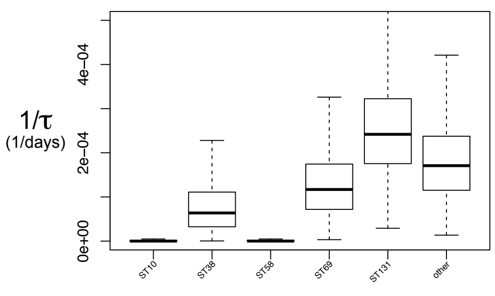

(c) Acquire carriership

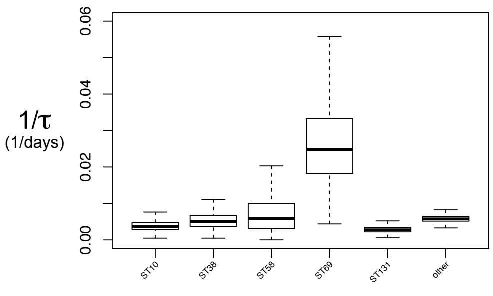

(d) Lose carriership

Estimates of the rates for state change $0 \to 1$ (acquire carriership) and $1 \to 0$ (lose carriership) by ESBL/pAmpC positive – MLST type, among the longitudinal study participants.
