# Supplementary material for: Time to acquire and lose carriership of ESBL/pAmpC producing E. coli in humans in the Netherlands
Source: PLoS One. 2018 Mar 21;13(3):e0193834. doi: 10.1371/journal.pone.0193834 (PMC5862452; doi:10.1371/journal.pone.0193834)
Supplement: S6 Fig — (PDF) [file pone.0193834.s006.pdf]

**S6 Fig. Waiting time distributions: losing carriership by *E. coli* MLST type**

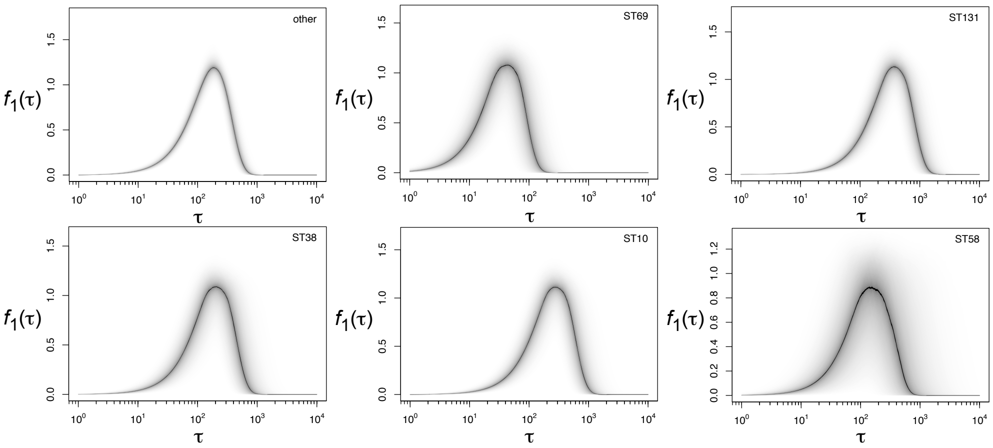


Distribution of the waiting time for state change $1 \rightarrow 0$ (lose carriership) by *E. coli* MLST type, positive for any ESBL/pAmpC gene.
