# Supplementary material for: Time to acquire and lose carriership of ESBL/pAmpC producing E. coli in humans in the Netherlands
Source: PLoS One. 2018 Mar 21;13(3):e0193834. doi: 10.1371/journal.pone.0193834 (PMC5862452; doi:10.1371/journal.pone.0193834)
Supplement: S7 Fig — (PDF) [file pone.0193834.s007.pdf]

S7 Fig. Parameter contours

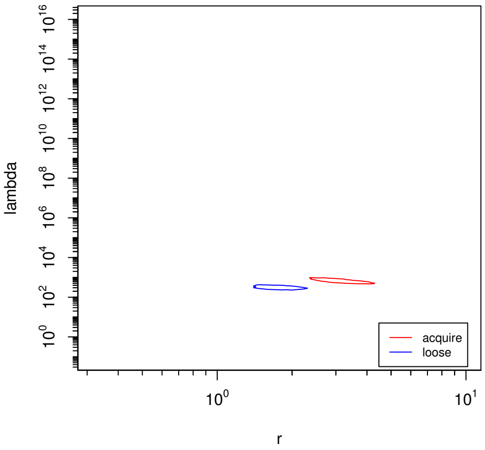

(e) Carriage of any ESBL/pAmpC gene

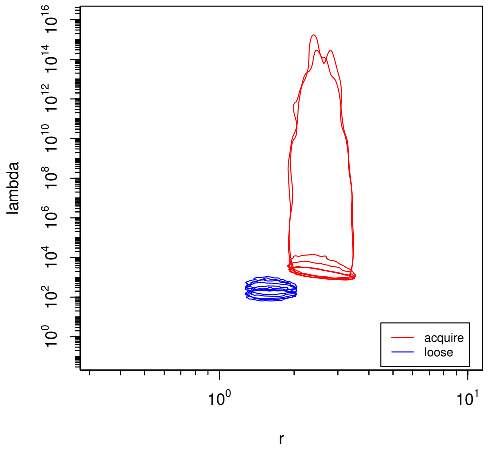

(f) By ESBL/pAmpC gene

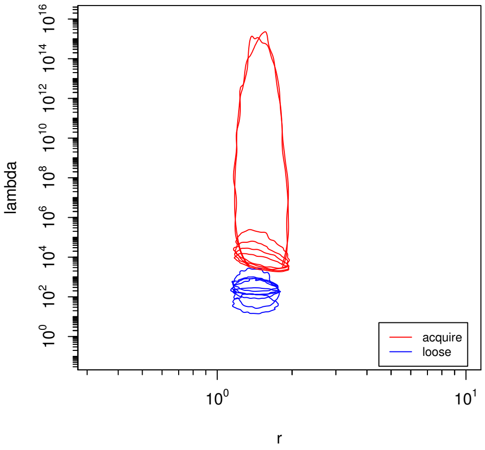

(g) By MLST type

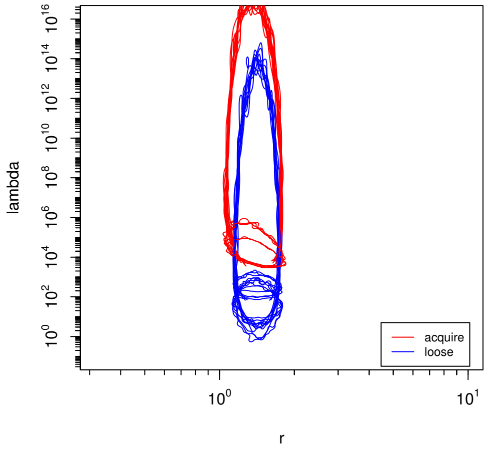

(h) By ESBL/pAmpC gene and MLST type

Distribution of the two Weibull parameters: 95% contour of the posterior sample, for state change $0 \to 1$ (acquire carriership) and $1 \to 0$ (lose carriership).
