# Supplementary material for: Time to acquire and lose carriership of ESBL/pAmpC producing E. coli in humans in the Netherlands
Source: PLoS One. 2018 Mar 21;13(3):e0193834. doi: 10.1371/journal.pone.0193834 (PMC5862452; doi:10.1371/journal.pone.0193834)
Supplement: S1 Table — (PDF) [file pone.0193834.s008.pdf]

**S1 Table. The 17 resistance genes found in the study.**

| ESBL/pAmpC genes | | | |
|---|---|---|---|
| $bla_{\text{CTX-M-1}}$ | $bla_{\text{CTX-M-2}}$ | $bla_{\text{CTX-M-3}}$ | $bla_{\text{CTX-M-8}}$ |
| $bla_{\text{CTX-M-14}}$ | $bla_{\text{CTX-M-15}}$ | $bla_{\text{CTX-M-27}}$ | $bla_{\text{CTX-M-32}}$ |
| $bla_{\text{CTX-M-55/57}}$ | $bla_{\text{CTX-M-65}}$ | $bla_{\text{CTX-M-104}}$ | $bla_{\text{CMY-119}}$ |
| $bla_{\text{CMY-2}}$ | $bla_{\text{DHA-1}}$ | $bla_{\text{SHV-2}}$ | $bla_{\text{SHV-12}}$ |
| $bla_{\text{TEM52}}$ | | | |
