# Supplementary material for: Time to acquire and lose carriership of ESBL/pAmpC producing E. coli in humans in the Netherlands
Source: PLoS One. 2018 Mar 21;13(3):e0193834. doi: 10.1371/journal.pone.0193834 (PMC5862452; doi:10.1371/journal.pone.0193834)
Supplement: S2 Table — (PDF) [file pone.0193834.s009.pdf]

S2 Table. MLST types

MLST types							
ST4	ST10	ST12	ST34	ST38	ST40	ST43	ST44
ST45	ST48	ST58	ST69	ST70	ST73	ST88	ST93
ST95	ST106	ST117	ST120	ST127	ST131	ST155	ST162
ST218	ST221	ST224	ST226	ST227	ST295	ST322	ST349
ST354	ST357	ST361	ST393	ST394	ST398	ST399	ST405
ST410	ST429	ST448	ST501	ST540	ST616	ST617	ST636
ST641	ST648	ST665	ST675	ST683	ST701	ST752	ST767
ST902	ST963	ST993	ST998	ST1001	ST1114	ST1122	ST1125
ST1140	ST1147	ST1193	ST1250	ST1276	ST1277	ST1288	ST1308
ST1431	ST1485	ST1589	ST1611	ST1656	ST1715	ST1844	ST1880
ST2076	ST2141	ST2178	ST2451	ST2459	ST2485	ST2539	ST3036
ST3052	ST3727	ST4373	ST4418	ST5017	ST5420	New	

The 94 MLST types found in the study. In addition to known *E. coli* types, MLST types not previously identified, were found (designated “New”).
