# Supplementary material for: Time to acquire and lose carriership of ESBL/pAmpC producing E. coli in humans in the Netherlands
Source: PLoS One. 2018 Mar 21;13(3):e0193834. doi: 10.1371/journal.pone.0193834 (PMC5862452; doi:10.1371/journal.pone.0193834)
Supplement: S3 Table — (PDF) [file pone.0193834.s010.pdf]

S3 Table. Gene/strain combinations

ESBL/pAmpC gene	MLST type	ESBL/pAmpC gene	MLST type	ESBL/pAmpC gene	MLST type
$bla_{\text{CTX-M-1}}$	ST10	$bla_{\text{CTX-M-15}}$	ST10	$bla_{\text{CTX-M-27}}$	ST131
$bla_{\text{CTX-M-1}}$	ST58	$bla_{\text{CTX-M-15}}$	ST38	$bla_{\text{CMY-2}}$	ST10
$bla_{\text{CTX-M-1}}$	ST69	$bla_{\text{CTX-M-15}}$	ST58	$bla_{\text{CMY-2}}$	ST38
$bla_{\text{CTX-M-14}}$	ST10	$bla_{\text{CTX-M-15}}$	ST131	$bla_{\text{CMY-2}}$	ST69
$bla_{\text{CTX-M-14}}$	ST38	$bla_{\text{CTX-M-27}}$	ST10	$bla_{\text{SHV-12}}$	ST58
$bla_{\text{CTX-M-14}}$	ST69	$bla_{\text{CTX-M-27}}$	ST38	$bla_{\text{SHV-12}}$	ST69
$bla_{\text{CTX-M-14}}$	ST131	$bla_{\text{CTX-M-27}}$	ST58		

Combinations of ESBL/pAmpC gene and MLST type for which rates were estimated.
