# Supplementary material for: Time to acquire and lose carriership of ESBL/pAmpC producing E. coli in humans in the Netherlands
Source: PLoS One. 2018 Mar 21;13(3):e0193834. doi: 10.1371/journal.pone.0193834 (PMC5862452; doi:10.1371/journal.pone.0193834)
Supplement: S4 Table — (PDF) [file pone.0193834.s011.pdf]

S4 Table. Parameter estimates by gene/strain: acquire ESBL-E

		r			λ (days)			$E(t)$ (days)		
		P_{50}	$P_{2.5}$	$P_{97.5}$	P_{50}	$P_{2.5}$	$P_{97.5}$	P_{50}	$P_{2.5}$	$P_{97.5}$
By ESBL/pAmpC gene and bacterial host strain acquire carriership										
$bla_{\text{CTX-M-1}}$	ST10	0.8	0.6	1.1	$5.8{\times}10^{9}$	$5.8{\times}10^{5}$	$2.0{\times}10^{17}$	$6.5{\times}10^{9}$	$6.1{\times}10^{5}$	$2.2{\times}10^{17}$
$bla_{\text{CTX-M-1}}$	ST58	0.8	0.6	1.1	$6.1{\times}10^{9}$	$5.0{\times}10^{5}$	$3.3{\times}10^{17}$	$7.0{\times}10^{9}$	$5.2{\times}10^{5}$	$3.8{\times}10^{17}$
$bla_{\text{CTX-M-1}}$	ST69	0.8	0.6	1.1	$8.0{\times}10^{9}$	$5.6{\times}10^{5}$	$3.2{\times}10^{17}$	$9.1{\times}10^{9}$	$6.0{\times}10^{5}$	$3.6{\times}10^{17}$
$bla_{\text{CTX-M-14}}$	ST10	0.8	0.6	1.1	$8.9{\times}10^{9}$	$4.3{\times}10^{5}$	$9.4{\times}10^{17}$	$10.1{\times}10^{9}$	$4.5{\times}10^{5}$	$10.7{\times}10^{17}$
$bla_{\text{CTX-M-14}}$	ST38	0.8	0.6	1.1	$6.6{\times}10^{5}$	$4.5{\times}10^{4}$	$1.6{\times}10^{8}$	$7.5{\times}10^{5}$	$4.6{\times}10^{4}$	$2.1{\times}10^{8}$
$bla_{\text{CTX-M-14}}$	ST69	0.8	0.6	1.1	$6.8{\times}10^{5}$	$4.7{\times}10^{4}$	$1.4{\times}10^{8}$	$7.7{\times}10^{5}$	$5.0{\times}10^{4}$	$1.8{\times}10^{8}$
$bla_{\text{CTX-M-14}}$	ST131	0.8	0.6	1.1	$2.2{\times}10^{5}$	$2.7{\times}10^{4}$	$8.0{\times}10^{6}$	$2.4{\times}10^{5}$	$2.7{\times}10^{4}$	$10.7{\times}10^{6}$
$bla_{\text{CTX-M-15}}$	ST10	0.8	0.6	1.1	$7.8{\times}10^{9}$	$4.9{\times}10^{5}$	$2.7{\times}10^{17}$	$8.9{\times}10^{9}$	$5.3{\times}10^{5}$	$2.7{\times}10^{17}$
$bla_{\text{CTX-M-15}}$	ST38	0.8	0.6	1.1	$8.1{\times}10^{9}$	$4.5{\times}10^{5}$	$3.5{\times}10^{17}$	$9.5{\times}10^{9}$	$4.8{\times}10^{5}$	$4.5{\times}10^{17}$
$bla_{\text{CTX-M-15}}$	ST58	0.8	0.6	1.1	$6.3{\times}10^{9}$	$5.5{\times}10^{5}$	$2.7{\times}10^{17}$	$7.3{\times}10^{9}$	$5.7{\times}10^{5}$	$3.2{\times}10^{17}$
$bla_{\text{CTX-M-15}}$	ST131	0.8	0.6	1.1	$6.8{\times}10^{5}$	$4.4{\times}10^{4}$	$1.5{\times}10^{8}$	$7.6{\times}10^{5}$	$4.4{\times}10^{4}$	$2.2{\times}10^{8}$
$bla_{\text{CTX-M-27}}$	ST10	0.8	0.6	1.1	$7.4{\times}10^{9}$	$6.0{\times}10^{5}$	$1.8{\times}10^{17}$	$8.3{\times}10^{9}$	$6.4{\times}10^{5}$	$2.3{\times}10^{17}$
$bla_{\text{CTX-M-27}}$	ST38	0.8	0.6	1.1	$5.4{\times}10^{9}$	$4.8{\times}10^{5}$	$1.6{\times}10^{17}$	$6.1{\times}10^{9}$	$5.0{\times}10^{5}$	$1.9{\times}10^{17}$
$bla_{\text{CTX-M-27}}$	ST58	0.8	0.6	1.1	$8.0{\times}10^{9}$	$4.6{\times}10^{5}$	$1.8{\times}10^{17}$	$8.8{\times}10^{9}$	$4.8{\times}10^{5}$	$2.0{\times}10^{17}$
$bla_{\text{CTX-M-27}}$	ST131	0.8	0.6	1.1	$2.2{\times}10^{5}$	$2.6{\times}10^{4}$	$0.7{\times}10^{8}$	$2.5{\times}10^{5}$	$2.5{\times}10^{4}$	$0.9{\times}10^{8}$
$bla_{\text{CMY-2}}$	ST10	0.8	0.6	1.1	$7.7{\times}10^{9}$	$6.2{\times}10^{5}$	$1.7{\times}10^{17}$	$8.6{\times}10^{9}$	$6.8{\times}10^{5}$	$2.2{\times}10^{17}$
$bla_{\text{CMY-2}}$	ST38	0.8	0.6	1.1	$7.8{\times}10^{9}$	$4.1{\times}10^{5}$	$3.4{\times}10^{17}$	$8.9{\times}10^{9}$	$4.6{\times}10^{5}$	$3.8{\times}10^{17}$
$bla_{\text{CMY-2}}$	ST69	0.8	0.6	1.1	$9.7{\times}10^{9}$	$4.7{\times}10^{5}$	$4.0{\times}10^{17}$	$11.1{\times}10^{9}$	$5.1{\times}10^{5}$	$4.4{\times}10^{17}$
$bla_{\text{SHV-12}}$	ST58	0.8	0.6	1.1	$6.7{\times}10^{9}$	$5.2{\times}10^{5}$	$8.9{\times}10^{17}$	$7.3{\times}10^{9}$	$5.5{\times}10^{5}$	$10.5{\times}10^{17}$
$bla_{\text{SHV-12}}$	ST69	0.8	0.6	1.1	$6.7{\times}10^{5}$	$4.3{\times}10^{4}$	$1.3{\times}10^{8}$	$7.6{\times}10^{5}$	$4.3{\times}10^{4}$	$1.7{\times}10^{8}$

Parameter estimates for the Weibull distributions of times to acquire carriership, by ESBL/pAmpC gene and *E. coli* MLST type. The shape parameter r is assumed the same for all strata; the estimates of the scale parameter λ are stratified by ESBL/pAmpC gene/MLST type combinations (S3: Table). Also shown is the mean time to acquire carriership ($E(\tau)$). For all estimates, median values (P_{50}) and 95% predictive ranges ($P_{2.5}$ – $P_{97.5}$) are given, to illustrate uncertainty.
