# Supplementary material for: Time to acquire and lose carriership of ESBL/pAmpC producing E. coli in humans in the Netherlands
Source: PLoS One. 2018 Mar 21;13(3):e0193834. doi: 10.1371/journal.pone.0193834 (PMC5862452; doi:10.1371/journal.pone.0193834)
Supplement: S5 Table — (PDF) [file pone.0193834.s012.pdf]

S5 Table. Parameter estimates by gene/strain: lose ESBL-E

		r			λ (days)			$E(t)$ (days)		
		P_{50}	$P_{2.5}$	$P_{97.5}$	P_{50}	$P_{2.5}$	$P_{97.5}$	P_{50}	$P_{2.5}$	$P_{97.5}$
By ESBL/pAmpC gene and bacterial host strain										
lose carriership										
$bla_{\text{CTX-M-1}}$	ST10	1.1	0.9	1.3	0.5×10^{2}	6.0×10^{0}	1.4×10^{3}	0.5×10^{2}	6.1×10^{0}	1.5×10^{3}
$bla_{\text{CTX-M-1}}$	ST58	1.1	0.9	1.3	0.2×10^{2}	1.2×10^{0}	0.9×10^{3}	0.2×10^{2}	1.2×10^{0}	0.8×10^{3}
$bla_{\text{CTX-M-1}}$	ST69	1.1	0.9	1.3	1.6×10^{5}	1.8×10^{1}	2.3×10^{12}	1.5×10^{5}	1.7×10^{1}	2.5×10^{12}
$bla_{\text{CTX-M-14}}$	ST10	1.1	0.9	1.3	4.2×10^{2}	1.3×10^{2}	3.2×10^{3}	4.2×10^{2}	1.3×10^{2}	3.2×10^{3}
$bla_{\text{CTX-M-14}}$	ST38	1.1	0.9	1.3	2.0×10^{2}	0.8×10^{2}	0.9×10^{3}	1.9×10^{2}	0.8×10^{2}	0.9×10^{3}
$bla_{\text{CTX-M-14}}$	ST69	1.1	0.9	1.3	0.7×10^{2}	0.1×10^{2}	1.8×10^{3}	0.7×10^{2}	0.1×10^{2}	1.8×10^{3}
$bla_{\text{CTX-M-14}}$	ST131	1.1	0.9	1.3	9.3×10^{5}	4.4×10^{2}	1.3×10^{13}	9.0×10^{5}	4.4×10^{2}	1.3×10^{13}
$bla_{\text{CTX-M-15}}$	ST10	1.1	0.9	1.3	9.8×10^{5}	4.0×10^{2}	1.5×10^{13}	9.6×10^{5}	3.9×10^{2}	1.5×10^{13}
$bla_{\text{CTX-M-15}}$	ST38	1.1	0.9	1.3	3.8×10^{5}	0.6×10^{2}	1.5×10^{13}	3.7×10^{5}	0.6×10^{2}	1.5×10^{13}
$bla_{\text{CTX-M-15}}$	ST58	1.1	0.9	1.3	3.3×10^{5}	0.6×10^{2}	0.6×10^{13}	3.2×10^{5}	0.6×10^{2}	0.6×10^{13}
$bla_{\text{CTX-M-15}}$	ST131	1.1	0.9	1.3	5.9×10^{2}	2.3×10^{2}	2.5×10^{3}	5.8×10^{2}	2.3×10^{2}	2.5×10^{3}
$bla_{\text{CTX-M-27}}$	ST10	1.1	0.9	1.3	2.8×10^{5}	0.6×10^{2}	0.5×10^{13}	2.8×10^{5}	0.5×10^{2}	0.5×10^{13}
$bla_{\text{CTX-M-27}}$	ST38	1.1	0.9	1.3	3.7×10^{5}	0.7×10^{2}	0.6×10^{13}	3.7×10^{5}	0.7×10^{2}	0.7×10^{13}
$bla_{\text{CTX-M-27}}$	ST58	1.1	0.9	1.3	3.6×10^{5}	0.5×10^{2}	0.6×10^{13}	3.5×10^{5}	0.5×10^{2}	0.5×10^{13}
$bla_{\text{CTX-M-27}}$	ST131	1.1	0.9	1.3	3.6×10^{2}	1.1×10^{2}	2.3×10^{3}	3.5×10^{2}	1.1×10^{2}	2.2×10^{3}
$bla_{\text{CMY-2}}$	ST10	1.1	0.9	1.3	9.9×10^{5}	3.4×10^{2}	1.9×10^{13}	9.6×10^{5}	3.4×10^{2}	1.8×10^{13}
$bla_{\text{CMY-2}}$	ST38	1.1	0.9	1.3	3.9×10^{5}	0.5×10^{2}	0.7×10^{13}	3.8×10^{5}	0.5×10^{2}	0.7×10^{13}
$bla_{\text{CMY-2}}$	ST69	1.1	0.9	1.3	0.3×10^{2}	0.1×10^{2}	1.0×10^{3}	0.3×10^{2}	0.1×10^{2}	1.0×10^{3}
$bla_{\text{SHV-12}}$	ST58	1.1	0.9	1.3	2.0×10^{5}	0.4×10^{2}	0.4×10^{13}	2.0×10^{5}	0.4×10^{2}	0.4×10^{13}
$bla_{\text{SHV-12}}$	ST69	1.1	0.9	1.3	0.6×10^{2}	0.1×10^{2}	0.5×10^{3}	0.6×10^{2}	0.1×10^{2}	0.5×10^{3}

Parameter estimates for the Weibull distributions of times to lose carriership, by ESBL/pAmpC gene and *E. coli* MLST type. The shape parameter r is assumed the same for all strata; the estimates of the scale parameter λ are stratified by ESBL/pAmpC gene/MLST type combinations (S3: Table). Also shown is the mean time to lose carriership ($E(\tau)$). For all estimates, median values (P_{50}) and 95% predictive ranges ($P_{2.5}$ – $P_{97.5}$) are given, to illustrate uncertainty.
