# Supplementary material for: Time to acquire and lose carriership of ESBL/pAmpC producing E. coli in humans in the Netherlands
Source: PLoS One. 2018 Mar 21;13(3):e0193834. doi: 10.1371/journal.pone.0193834 (PMC5862452; doi:10.1371/journal.pone.0193834)
Supplement: S1 Appendix — (PDF) [file pone.0193834.s013.pdf]

S1 Appendix. Independence of ESBL/pAmpC genes and *E. coli* MLST types

In case the average rate of acquiring a specific gene is λ_g, the probability that a person becomes a carrier in a period $(0, \tau)$ is

$$P_g = 1 - \mathrm{e}^{-\lambda_g \tau} \approx \lambda_g \tau$$

the latter approximation holds when τ is small. When the average rate of acquiring a specific *E. coli* strain is λ_s, the probability of acquiring that strain would be proportional to λ_s. However, any *E. coli* would only be identified when it carried a ESBL/pAmpC gene. Therefore, this probability of acquiring a strain is conditional on any gene being present, with probability $P_g^* \approx \sum \lambda_g \tau$. The summation includes all genes found in the study. If genes occur independently (i.e. presence of one gene variant does not influence the probability that another variant is also present), the probability of finding a specific *E.coli* strain may be

$$P_s = \frac{\lambda_s}{\sum \lambda_g} \tau$$

When we have an estimate of the average rate of acquiring carriership for a specific combination of ESBL/pAmpC gene and *E. coli* strain, λ_{sg}, and acquiring a gene and a strain would be independent, then

$$\lambda_{sg} = \frac{\lambda_s}{\sum \lambda_g} \lambda_g$$

Which can be checked for the combinations of genes and *E. coli* strains analyzed in this study. A similar argument may be given for losing genes/strains.

Thus, it may be tested whether the rate of acquiring or losing a combination of resistance gene and *E. coli* strain is equal to the product of acquiring the gene and the *E. coli* strain separately. This may provide an indication whether resistance genes would be acquired or lost independently of the *E. coli* strain that host the plasmids they reside on.

Using this crude approximation, it appears that when acquiring carriership of ESBL/pAmpC resistance, gene and *E. coli* strain are independent (Table below). However, when losing carriership, for several combinations the rate of losing the

combination is smaller than the product of the separate rates for the same gene and *E. coli* strain. Those combination then would be more persistant than expected from the separate rate estimates.

Table: Testing independence of acquiring or losing ESBL/pAmpC genes and *E. coli* MLST types. Fraction positives (∗ indicates a fraction greater than 0.95 or smaller than 0.05).

ESBL/pAmpC gene	*E. coli* MLST type	acquire Fraction +	acquire signif.	lose Fraction +	lose signif.
$bla_{\text{CTX-M-1}}$	ST10	0.51	–	1	∗∗
$bla_{\text{CTX-M-1}}$	ST58	0.51	–	1	∗∗
$bla_{\text{CTX-M-1}}$	ST69	0.95	–	0.34	–
$bla_{\text{CTX-M-14}}$	ST10	0.52	–	1	∗∗
$bla_{\text{CTX-M-14}}$	ST38	0.22	–	1	∗∗
$bla_{\text{CTX-M-14}}$	ST69	0.35	–	0.99	∗
$bla_{\text{CTX-M-14}}$	ST131	0.33	–	0.43	–
$bla_{\text{CTX-M-15}}$	ST10	0.54	–	0.39	–
$bla_{\text{CTX-M-15}}$	ST38	0.94	–	0.42	–
$bla_{\text{CTX-M-15}}$	ST58	0.53	–	0.41	–
$bla_{\text{CTX-M-15}}$	ST131	0.60	–	1	∗∗
$bla_{\text{CTX-M-27}}$	ST10	0.52	–	0.43	–
$bla_{\text{CTX-M-27}}$	ST38	0.93	–	0.42	–
$bla_{\text{CTX-M-27}}$	ST58	0.52	–	0.41	–
$bla_{\text{CTX-M-27}}$	ST131	0.31	–	1	∗∗
$bla_{\text{CMY-2}}$	ST10	0.23	–	0.35	–
$bla_{\text{CMY-2}}$	ST38	0.49	–	0.42	–
$bla_{\text{CMY-2}}$	ST69	0.52	–	0.99	∗
$bla_{\text{SHV-12}}$	ST58	0.50	–	0.36	–
$bla_{\text{SHV-12}}$	ST69	0.20	–	1	∗∗
