# Supplementary material for: Time to acquire and lose carriership of ESBL/pAmpC producing E. coli in humans in the Netherlands
Source: PLoS One. 2018 Mar 21;13(3):e0193834. doi: 10.1371/journal.pone.0193834 (PMC5862452; doi:10.1371/journal.pone.0193834)
Supplement: S2 Appendix — (PDF) [file pone.0193834.s014.pdf]

S2 Appendix. R code for construction of the likelihood function

Sample R code for construction of the likelihood function, to be used for MCMC sampling.

```
trange <- c(min(tobs,na.rm=TRUE),max(tobs,na.rm=TRUE));
n.subj <- nrow(sobs); n.obs <- c();
for(k.subj in 1:n.subj){
  len <- length(sobs[k.subj,]);
  while(is.na(sobs[k.subj,len])) len <- len - 1;
  n.obs <- c(n.obs,len);
}
ind.l <- array(NA,dim=dim(sobs)); # subject, state
tm <- array(NA,dim=c(n.subj,3,ncol(sobs)-1)); # subject, t1/t2- start/end, number
for(k.subj in 1:n.subj){
  k.obs <- 1; k.l <- 1; # 1st observation for this subject
  while(is.na(sobs[k.subj,k.obs])) k.obs <- k.obs + 1; # skip missing obs.
  old.st <- k.obs; # current observation
  ind.l[k.subj,k.l] <- sobs[k.subj,old.st] + 1; # status at current obs.
  k.tm <- 1;
  while(k.obs < n.obs[k.subj]){ # penultimate observation for this subject?
    k.obs <- k.obs + 1; # next observation
    while(is.na(sobs[k.subj,k.obs])) k.obs <- k.obs + 1; # skip missing obs.
    new.st <- k.obs; # new observation
    if(sobs[k.subj,old.st]!=sobs[k.subj,new.st]) k.l <- k.l + 1; # state change?
    ind.l[k.subj,k.l] <- sobs[k.subj,new.st] + 1; # update state
    if(sobs[k.subj,old.st]==0 & sobs[k.subj,new.st]==1){ # 0 -> 1 transition?
      tm[k.subj,,k.tm] <- c(1,old.st,new.st); k.tm <- k.tm + 1; # new t1
    }
    if(sobs[k.subj,old.st]==1 & sobs[k.subj,new.st]==0){ # 1 -> 0 transition?
      tm[k.subj,,k.tm] <- c(2,old.st,new.st); k.tm <- k.tm + 1; # new t2
    }
    old.st <- new.st; # update previous state to current one
  }
}

ind.tm <- array(NA,dim=c(n.subj,dim(tm)[3]*2));
cens.lev <- array(NA,dim=c(length(which(!is.na(tm)))/3,2));
k.obs <- 1;
for(k.subj in 1:n.subj){
  if(!all(is.na(tm[k.subj,1,]))){
```

```
    k.t <- 1; k.tm <- 1;
    ind.tm[k.subj,k.tm] <- k.obs;
    cens.lev[k.obs,] <- tobs[k.subj,tm[k.subj,2:3,k.t]];
    k.tm <- k.tm + 1; k.t <- k.t + 1; k.obs <- k.obs + 1;
    while(k.t <= length(which(!is.na(tm[k.subj,1,])))){
      cens.lev[k.obs,] <- tobs[k.subj,tm[k.subj,2:3,k.t]];
      ind.tm[k.subj,k.tm] <- k.obs;
      k.tm <- k.tm + 1;
      ind.tm[k.subj,k.tm] <- k.obs - 1;
      k.tm <- k.tm + 1;
      k.obs <- k.obs + 1;
      k.t <- k.t + 1;
    }
    ind.tm[k.subj,k.tm] <- k.obs - 1;
  }
}

n.l <- rep(NA,n.subj); n.t <- rep(NA,n.subj);
for(k.subj in 1:n.subj){
  n.l[k.subj] <- length(which(!is.na(ind.l[k.subj,])));
  n.t[k.subj] <- length(which(!is.na(ind.tm[k.subj,])));
}
num.t <- max(ind.tm,na.rm=TRUE);
subj.1 <- (1:n.subj)[n.l > 1];
subj.2 <- (1:n.subj)[n.l == 1];
```
