# Supplementary material for: Time to acquire and lose carriership of ESBL/pAmpC producing E. coli in humans in the Netherlands
Source: PLoS One. 2018 Mar 21;13(3):e0193834. doi: 10.1371/journal.pone.0193834 (PMC5862452; doi:10.1371/journal.pone.0193834)
Supplement: S3 Appendix — (PDF) [file pone.0193834.s015.pdf]

## S3 Appendix. JAGS code for MCMC sampling of the posterior distribution

Sample JAGS code for obtaining a posterior sample of the parameters of the waiting time distributions for acquiring and losing carriership.

```
model{
  C <- 1000
  for(k.subj in subj.1){
    lik[k.subj,1] <-
      exp(-((t[ind.t[k.subj,1]]-tobs[k.subj,1])/
        lambda[ind.l[k.subj,1]])^r[ind.l[k.subj,1]])
    for(k.lik in 2:(n.l[k.subj]-1)){
      lik[k.subj,k.lik] <- ifelse(n.l[k.subj] > 2,

        (r[ind.l[k.subj,k.lik]]/lambda[ind.l[k.subj,k.lik]])*
((t[ind.t[k.subj,2+2*(k.lik-2)]]-t[ind.t[k.subj,3+2*(k.lik-2)]])/
  lambda[ind.l[k.subj,k.lik]])^(r[ind.l[k.subj,k.lik]]-1)*
exp(-((t[ind.t[k.subj,2+2*(k.lik-2)]]-t[ind.t[k.subj,3+2*(k.lik-2)]])/
  lambda[ind.l[k.subj,k.lik]])^r[ind.l[k.subj,k.lik]]),1)
    }
    lik[k.subj,n.l[k.subj]] <-
      exp(-((tobs[k.subj,n.obs[k.subj]]-t[ind.t[k.subj,n.t[k.subj]]])/
        lambda[ind.l[k.subj,n.l[k.subj]]])^r[ind.l[k.subj,n.l[k.subj]]])
    loglik[k.subj] <- sum(log(lik[k.subj,(1:n.l[k.subj])]))
  }
  for(k.subj in subj.2){
    lik[k.subj,1] <-
      exp(-((tobs[k.subj,n.obs[k.subj]]-tobs[k.subj,1])/
        lambda[ind.l[k.subj,1]])^r[ind.l[k.subj,1]])
    loglik[k.subj] <- log(lik[k.subj,1])
  }
  for(k.t in 1:num.t){
    # tcens[k.t] ~ dinterval(t[k.t],censlev[k.t,])
    # t[k.t] ~ dunif(trange[1],trange[2])
    t[k.t] ~ dunif(censlev[k.t,1],censlev[k.t,2])

  }
  phi <- sum(loglik)
  zero ~ dpois(-phi+C)
  for(k.state in 1:2){
```

```
    r[k.state] <- exp(logr[k.state])
    lambda[k.state] <- exp(loglambda[k.state])
    logr[k.state] ~ dnorm(rprior[1],rprior[2])
    loglambda[k.state] ~ dnorm(lprior[k.state,1],lprior[k.state,2])
  }
}
```
